# HepG2-NTCP Subclones Exhibiting High Susceptibility to Hepatitis B Virus Infection

**DOI:** 10.3390/v14081800

**Published:** 2022-08-17

**Authors:** Muhammad Atif Zahoor, Adrian Kuipery, Alexander I. Mosa, Adam J. Gehring, Jordan J. Feld

**Affiliations:** 1 Toronto Center for Liver Disease, Toronto General Hospital Research Institute, University Health Network, Toronto, ON M5G 1L7, Canada; 2 Department of Immunology, University of Toronto, Toronto, ON M5S 1A8, Canada

**Keywords:** covalently closed circular DNA (cccDNA), hepatitis B virus, HepG2-NTCP cells, immunofluorescence, sodium taurocholate co-transporting polypeptide (NTCP) receptor, subcloning, limiting dilution, Myrcludex B

## Abstract

HepG2 cells reconstituted with Hepatitis B virus (HBV) entry receptor sodium taurocholate co-transporting polypeptide (NTCP) are widely used as a convenient in vitro cell culture infection model for HBV replication studies. As such, it is pertinent that HBV infectivity is maintained at steady-state levels for an accurate interpretation of in vitro data. However, variations in the HBV infection efficiency due to imbalanced NTCP expression levels in the HepG2 cell line may affect experimental results. In this study, we performed single cell-cloning of HepG2-NTCP-A3 parental cells via limiting dilution and obtained multiple subclones with increased permissiveness to HBV. Specifically, one subclone (HepG2-NTCP-A3/C2) yielded more than four-fold higher HBV infection compared to the HepG2-NTCP-A3 parental clone. In addition, though HBV infectivity was universally reduced in the absence of polyethylene glycol (PEG), subclone C2 maintained relatively greater permissiveness under PEG-free conditions, suggesting the functional heterogeneity within parental HepG2-NTCP-A3 may be exploitable in developing a PEG-free HBV infection model. The increased viral production correlated with increased intracellular viral antigen expression as evidenced through HBcAg immunofluorescence staining. Further, these subclones were found to express different levels of NTCP, albeit with no remarkable morphology or cell growth differences. In conclusion, we isolated the subclones of HepG2-NTCP-A3 which support efficient HBV production and thus provide an improved in vitro HBV infection model.

## 1. Introduction

Hepatitis B virus (HBV) infection is a major health problem worldwide, with 3.5% of the global population chronically infected. Though infections in adulthood are typically acute and self-resolving, chronic infection is characterized by persistent, asymptomatic liver inflammation, leading to progressive fibrosis that may culminate in severe complications, such as cirrhosis and hepatocellular carcinoma [1,2]. Although HBV is vaccine-preventable and current therapy with nucleos(t)ide analogues (NUCs) can suppress virus replication, the treatment is usually life-long and viral clearance is rare. Nearly one million people die every year due to HBV-related complications [3].

With narrow host and tissue specificity, in vitro models permissive to HBV are critical tools in understanding the viral replication and designing curative therapies. Though hepatoma lines have been used for decades to study HBV replication, recent advances using HepG2 cells reconstituted with the HBV receptor, sodium taurocholate co-transporting polypeptide (NTCP), have provided an accessible, standardized in vitro model for HBV infection [3,4,5,6]. We hypothesized that the subpopulations within this model would manifest variability in permissiveness to HBV. This is an important consideration in reducing inter- and intra-experimental variability, as the varying representation of subpopulations within a culture may skew the efficacy of candidate antivirals and susceptibility to infection. Further, the selective culturing of the subpopulations with increased permissiveness could increase the reproducibility and efficiency of the HepG2-NTCP in vitro model. In this study, we performed subcloning of the HepG2-NTCP-A3 parental clone and obtained subclones which exhibited variations in permissiveness to HBV infection. We isolated the most permissive line and observed an increased expression of NTCP relative to the parental line. Collectively, these findings suggest this subclone provides an improved HBV infection model, and that parental HepG2-NTCP subpopulations should be routinely screened to reduce intra-lineage variability in HBV permissiveness.

## 2. Materials and Methods

### 2.1. Generation of HepG2-NTCP Subclones

The HepG2 and HepG-NTCP-A3 cells were cultured in Dulbecco’s Modified Eagles Medium (DMEM; Sigma-Aldrich, Oakville, ON, Canada), supplemented with 10% fetal bovine serum (FBS) (GIBCO, Mississauga, ON, Canada), L-glutamine, 100 U penicillin/streptomycin Thermo Fisher Scientific, Mississauga, ON, Canada), 1 mM Sodium Pyruvate, 20 mM HEPES, essential and non-essential amino acids (Corning, Manasas, VA, USA) and 5 µg plasmosin (Invivogen, Cedarlane, Burlington, ON, Canada) at 37 °C in a 5% humidified incubator, as described [7]. The HepG2-NTCP-A3 subclones were obtained by limiting dilution, essentially as described [8]. Briefly, a total of 96 cells in 20 mL media containing 2.5 µg/mL puromycin were seeded at 200 uL per well into a 96 well plate. After two weeks, the single cell clones were isolated, expanded and stored at −196 °C until further use.

### 2.2. NTCP Expression

The HepG2-NTCP-A3 subclones were expanded and the total RNA was isolated, using the RNeasy mini kit (QIAgen, Toronto, ON, Canada). The RNA concentration was measured by nanodrop and subjected to cDNA synthesis, as described [9]. The amounts of NTCP and GAPDH mRNAs were estimated by quantitative real-time PCR, as described [10], with the Quant Studio 6 Flex system (Thermo Scientific, Mississauga, ON, Canada) using the primer pairs 5′-CTCTCTTCTGCCTCAATGGAC-3′ and 5′-CAGTTGTGGCAGCTGTGTAG-3′ and 5′-GAAGGTCGGAGTCAACGGATT-3′ and 5′-TGATGACAAGCTTCCCGTTCTC-3′, respectively.

### 2.3. Peptide Labelling and Staining of NTCP Expressing Cells

The Myrcludex B (MyrB), a lipopeptide consisting of amino acid residues 2–48 of the pre-S1 region of the HBV large surface antigen [11], was labelled with Alexa Fluor 647 protein-labeling kit, according to the manufacturer’s instructions (Thermo Scientific, Mississauga, ON, Canada), and designated as MyrB-Alexa-647. For the staining of the NTCP expression, HepG2, HepG2-NTCP-A3 parental clone or its subclones B7, C2, D10, G4 and G7 were seeded into a 12-well plate and incubated with 200 nM MyrB-Alexa-647 at 37 °C for 30 min, as described [12,13]. The unbound peptide was removed by washing with 1× PBS, the cells were fixed with 4% paraformaldehyde (PFA) and imaged using the EVOS FL-Auto 2 fluorescence microscope (Invitrogen, Burlington, ON, Canada), as described [7].

### 2.4. HBV Infection and Quantification of HBV Genomes

The HepG2-NTCP-A3 parental clone or its subclones were seeded at 500,000 cells per ml per well into a 12-well plate and infected with HepAD38-cell harvested HBV-genotype D at 100, 200, 500 or 1000 Genome Equivalents (GEq)/mL in the presence of 2.5% dimethyl sulfoxide (DMSO; Sigma-Aldrich, Oakville, ON, Canada) and 4% or no polyethylene glycol (PEG 8000; Sigma-Aldrich, Oakville, ON, Canada), as described [7,14,15,16]. The next day, the virus inoculum was removed, and the cells were extensively washed with PBS and cultured in the presence of DMSO-2.5%. Four days post-infection, the viral DNA was extracted from the supernatants using the DNeasy blood and tissue kit (QIAgen, Toronto, ON, Canada), according to the manufacturer’s instructions, and quantified using HBV 1844F 5′-GTTGCCCGTTTGTCCTCTAATTC-3′ as a forward and HBV 1745R 5′-GGAGGGATACATAGAGGTTCCTTGA-3′ as a reverse primer, as described [17], with the Quant Studio 6 Flex system (Thermo Scientific, Mississauga, ON, Canada). The HBV 1.3-mer plasmid DNA (Addgene # 6820) was used as a standard to calculate the amount of HBV DNA. With written informed consent and our institutional study ethics approval, the serum samples from the three chronically infected HBV patients with a viral load of 3.42 ± 2.11 × 10^8^ GEq/mL were obtained from the Toronto Center for Liver Disease, as described [18]. The DNA was extracted as described above, and the viral surface gene was amplified with 5′-TCACCATATTCTTGGGAACAAGA-3′ and 5′-CGAACCACTGAACAAATGGC-3′ as the forward and reverse primers, respectively. The PCR product was sequenced using 5′- TGGGAACAAGAGCTACAGCATGG-3′ as a sequencing primer and the genotypes were determined, using the NCBI genotyping online tool as described [19]. The crude and unpurified serum samples are known to reduce HBV infectivity [16]; therefore, the serum samples were buffer-exchanged using Amicon Ultra 0.5 µM filter units (Sigma, Oakville, ON, Canada) by centrifugation at 13,000 rpm for 30 min, as described [20], and used to infect the HepG2-NTCP-A3 parental and C2 subclone at 500 GEq/mL for 24 h. The next day, the virus inoculum was removed, and the infected cells were maintained for 7 days in the presence of 2.5% DMSO. The viral DNA was extracted from the cell culture supernatant, and quantified as described above. The quantification of the HBV cccDNA was performed by qPCR, as described [16,17,21,22]. Briefly, the cells were infected with HBV at various MOIs in the presence or absence of MyrB as a control (1 µM), as described [22]. The DNA was extracted from the infected cells using the DNeasy blood and tissue kit (QIAgen, Toronto, ON, Canada), as described above except that the cell lysates were incubated for 2 h at 56 °C. A total of 200 ng of DNA was used for the cccDNA quantification by real time PCR with p1040 5′-GTGGTTATCCTGCGTTGAT-3′ as a forward and p1996 5′GAGCTGAGGCGGTATCT-3′ as a reverse primer. The p1085 5′-FAM-AGTTGGCGAGAAAGTGAAAGCCTGC-TAMRA-3′ was used as a probe. The real time PCR was performed, using the TaqMan Mix on Studio 6 Flex system (Thermo Scientific, Mississauga, ON, Canada) with the following reaction conditions: 95 °C for 15 min; then 50 cycles of 95 °C for 5 s; and 63 °C for 70 s. The serial dilutions of pSHH2.1 plasmid were used as the quantification standards. The human β-globin was quantified with sybr green, using 5′-CAGGTACGGCTGTCATCACTTAGA-3′ and 5′-CATGGTGTCTGTTTGAGGTTGCTA-3′ as the forward and reverse primers, respectively, to normalize the cccDNA copies, as described [22].

### 2.5. Immunofluorescence for HBV Core Protein

The HepG2-NTCP-A3 parental clone or its subclones were cultured and infected with HBV, as described above. The cells were fixed in 4% PFA at room temperature, washed with PBS and permeabilized with 0.5% Triton X-100. After blocking, the cells were incubated with rabbit polyclonal HBV anti-Core antibody C149 at 1:5000 dilution, as described [23], as a primary antibody and donkey anti-rabbit Alexa fluor-647 at 1:500 dilution (Thermo Scientific, Mississauga, ON, Canada) as a secondary antibody. The nuclei were stained with DAPI, and the cells were imaged using EVOS FL Auto 2 (Invitrogen, Burlington, ON, Canada), as described [7].

### 2.6. Statistical Analysis

The analyses were carried out using GraphPad Prism (San Diego, CA, USA) as described [9]. The data are shown as mean ± SD.

## 3. Results

### 3.1. Enhanced HBV Infection in Subclones of HepG2-NTCP Cells

**Figure 1:**
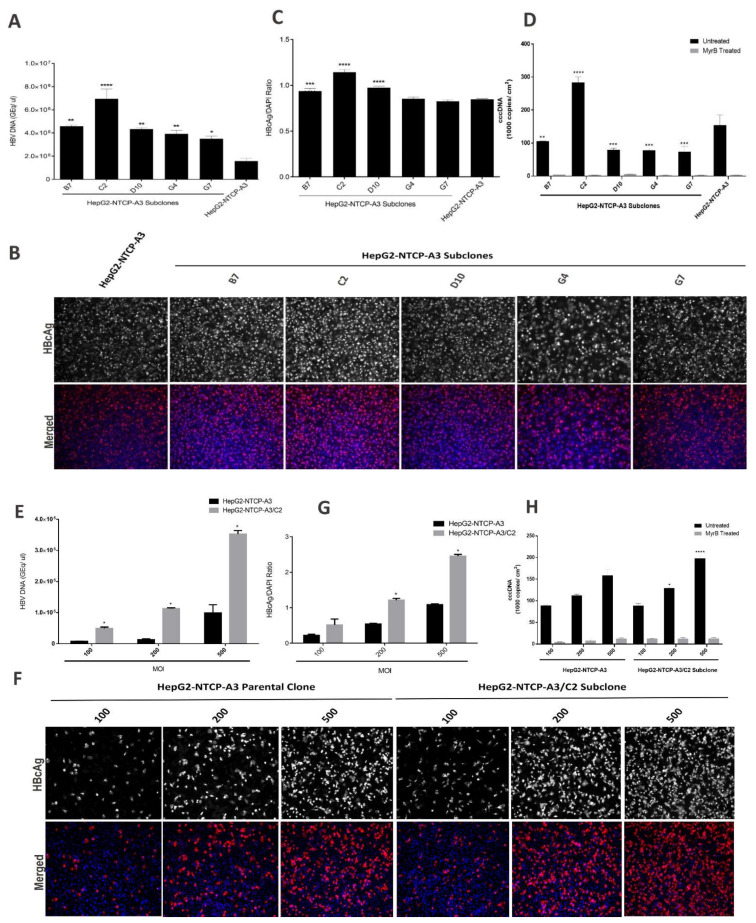
HBV infection in HepG2-NTCP-A3 subclones. (**A**). Indicated subclones of HepG2-NTCP-A3 were infected with HBV at 1000 GEq/mL in the presence of PEG8000; Cell culture supernatants were collected, and the amount of HBV was quantified by real-time PCR; (**B**). HepG2-NTCP-A3 subclones infected with HBV were fixed and immunostained with an antibody to HBcAg and counterstained with DAPI. The cells were visualized by fluorescence microscopy; (**C**). HBcAg fluorescence over DAPI shown as a ratio. Data are presented as mean ± SD (One way ANOVA; *p* < 0.0001; * level of significance); (**D**). HepG2-NTCP-A3 parental clone or its subclones were infected with HBV in the presence or absence of Myrcludex B (1 µM; MyrB). DNA was extracted from the infected cells after four days post infection and cccDNA copies relative to β-globin were quantified by real time PCR. Data are presented as mean ± SD (Two way ANOVA; *p* < 0.0001; * level of significance); (**E**). C2 subclone and HepG2-NTCP-A3 parental clone were infected with HBV at 100, 200 and 500 MOI. Supernatants were collected at day 4 post-infection for DNA quantification (**F**). The cells were fixed and immunostained with HBcAg antibodies and nuclei were counterstained with DAPI; (**G**). HBcAg fluorescence over DAPI shown as a ratio. Data are presented as mean ± SD (*t*-test; *p* < 0.005); (**H**). DNA from HepG2-NTCP-A3 or HepG2-NTCP-A3/C2 subclone infected with HBV in the presence or absence of MyrB at 100, 200 or 500 MOI was extracted and cccDNA copies relative to β-globin were quantified by real time PCR. Data are presented as mean ± SD (Two way ANOVA; *p* < 0.0001; * level of significance).

To obtain the subclones of the HepG2-NTCP-A3 with enhanced HBV infectivity, we performed subcloning through limiting dilution and obtained twelve subclones. Two of the clones showed irregular morphology and did not grow. We expanded five subclones designated as B7, C2, D10, G4 and G7, infected each with HBV overnight, with or without PEG8000, and then cultured them in growth media for an additional three days. The extracellular HBV DNA was assessed in the culture supernatant. The results showed that, following HBV infection in the presence of PEG8000, the subclones B7, C2, D10, G4 and G7 yielded 2.98, 4.45-, 2.8-, 2.57- and 2.25-fold significantly higher HBV infectivity than the parental HepG2-NTCP-A3 clone (Figure 1A; Table 1). These data suggested that subcloning improved the HBV permissiveness. Next, we performed IFA of the HBV-infected subclones and observed, in agreement with our qPCR data, that the HBV was more infectious in the HepG2-NTCP-A3 subclones (Figure 1B,C; Table 1). The cccDNA copies from the HBV-infected HepG2-NTCP-A3 or its subclones were quantified by qPCR. The results showed that, in the absence of MyrB, the cccDNA copies in all of the subclones were significantly different compared with the HepG2-NTCP-A3 parental clone. Consistent with the high susceptibility to HBV (Figure 1A,B), C2 was the only subclone which showed an approximately two-fold higher number of cccDNA copies than the HepG2-NTCP-A3 parental control (Figure 1D). All of the other subclones, however, showed a lower number of cccDNA copies than the HepG2-NTCP-A3 parental clone (Figure 1D).

Next, we selected the most permissive subclone C2 to determine if the enhanced infectivity was observed across the different multiplicities of infection (MOI). We found that C2 was more permissive than the parental clone across a range of MOI, with the greatest proportional and absolute increases at MOI of 200 and 500, respectively (Figure 1E–G). The cccDNA copies from the HepG2-NTCP-A3 or C2 subclone infected with HBV at 100, 200 or 500 MOI, were quantified by qPCR. The results showed that the cccDNA copies increased with increasing MOI in both the HepG2-NTCP-A3 parental and C2 subclone. Further, the C2 subclone showed a significantly higher number of cccDNA copies than the HepG2-NTCP-A3 parental control at 200 and 500 MOI (Figure 1H). Collectively, these data suggest that the C2 subclone showed significantly higher levels of viral DNA, a higher number of cccDNA copies and enhanced infectivity; however, the high MOI is still required for efficient HBV infectivity.

**Figure 2:**
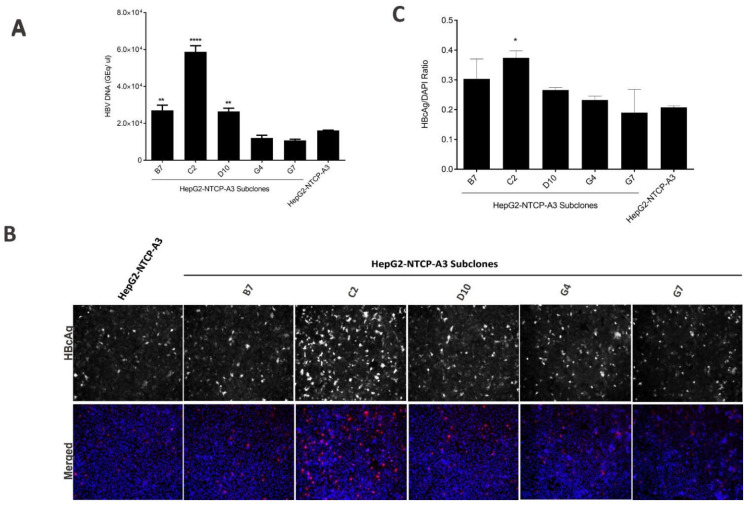
PEG-free HBV infection in HepG2-NTCP-A3 subclones. (**A**). Indicated subclones of HepG2-NTCP-A3 were infected with HBV in PEG-free conditions and the amount of HBV DNA in the cell culture supernatant was quantified by real-time qPCR; (**B**). PEG-free HBV infected cells were fixed and immunostained with HBcAg and counterstained with DAPI. The cells were visualized by fluorescence microscopy; (**C**). HBcAg fluorescence over DAPI shown as a ratio. Data are presented as mean ± SD (One way ANOVA; *p* < 0.0001, * level of significance).

To investigate PEG-free HBV infection efficiency, subclones B7, C2, D10, G4, G7 and the parental clone HepG2-NTCP-A3 were infected with HBV overnight in the absence of PEG. The extracellular HBV DNA was assessed in the culture supernatant. The results showed that, compared to 4% PEG8000 (Figure 1), the HBV infection was significantly lower in the PEG-free conditions (Figure 2A–C; Table 1). The cccDNA copies from HepG2-NTCP-A3 or its subclones infected with HBV under PEG-free conditions could not be quantified, and there was no significant difference between the MyrB-treated and untreated samples (data not shown) suggesting that higher infectivity is required for the cccDNA quantification. However, the subclone C2 maintained greater permissiveness to HBV infection in the PEG-free conditions than either the parental clone or the other subclones (Table 1), with a relative increase in infection efficiency under PEG-free conditions, suggesting the C2 subclone can be utilized if PEG-free conditions are required.

**Figure 3:**
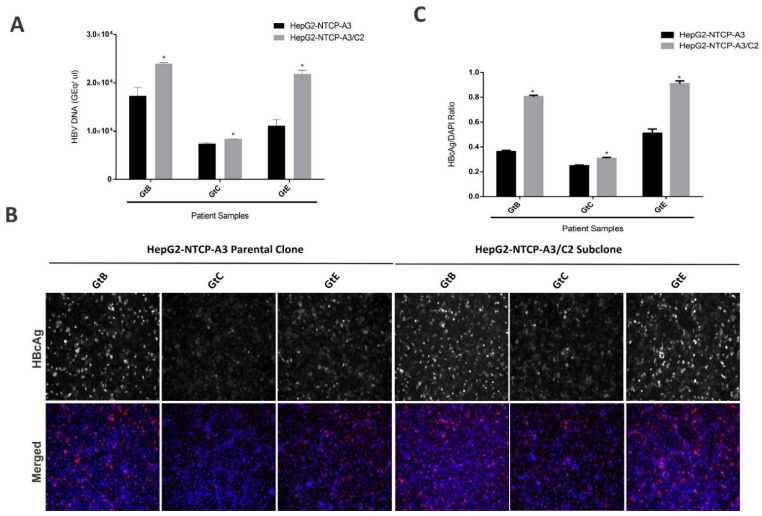
Susceptibility of C2 subclone to HBV Clinical isolates. (**A)** HepG2-NTCP-A3 parental clone and C2 subclone were infected with three sequence confirmed genotype B, C and E patient samples. Supernatants were collected at day 7 post-infection and the amount of HBV DNA was quantified by real-time PCR; (**B**). HBV infected cells were fixed and immunostained with an antibody to HBcAg and counterstained with DAPI. The cells were visualized by fluorescence microscopy; (**C**). HBcAg fluorescence over DAPI shown as a ratio. Data are presented as mean ± SD (*t*-test; *p* < 0.05, * level of significance).

To determine if the enhanced permissiveness of C2 was limited to a single HBV genotype, which would imply that the permissive phenotype was a property emergent from a genotype–subclone interaction, rather than an intrinsic feature of the subclone, we evaluated the infectivity using three patient-derived HBV isolates. Across the genotypes B, C, and E, we observed enhanced permissiveness in the C2 subclone, though the absolute increase in infectivity was variable across the genotypes (Figure 3A–C), suggesting that both the intrinsic and emergent properties contribute to C2′s increased permissiveness.

### 3.2. NTCP Expression in Subclones of HepG2-NTCP Cells

**Figure 4:**
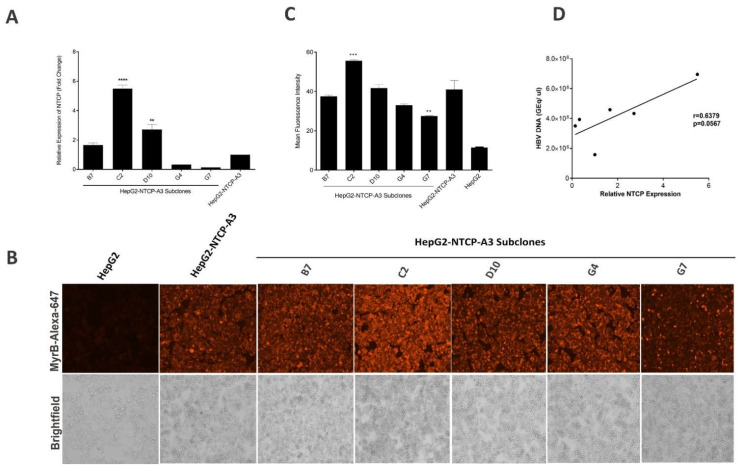
NTCP expression in subclones of HepG2-NTCP-A3 cells. (**A**) HepG2-NTCP-A3 parental clone stably expressing human NTCP were subcloned by limiting dilution into 96 well plates. Individual subclones were expanded and processed for NTCP mRNA quantification by reverse transcriptase real time PCR. HepG2-NTCP-A3 parental clone was set to 1 and represented as a fold change and the data are presented as mean ± SD (One way ANOVA; *p* < 0.0001; * level of significance); (**B**). HepG2, HepG2-NTCP-A3 parental clone and its subclones B7, C2, D10, G4 and G7 were stained for cell surface NTCP expression using Alexa Fluor labelled Myrcludex B (MyrB-Alexa-647) and visualized by fluorescence microscopy; (**C**). Quantitative representation of cell surface NTCP expression as mean fluorescence intensity (MFI); (**D**). Pearson correlation coefficient between NTCP expression levels and HBV infection in HepG2-NTCP-A3 parental clone and its subclones B7, C2, D10, G4 and G7.

To evaluate if the NTCP expression varied between the most permissive subclones and the parental lineage, the subclones B7, C2, D10, G4 and G7 and HepG2-NTCP-A3 were expanded, and the NTCP mRNA expression levels were analyzed by qRT-PCR. The results showed that the B7, C2 and D10 subclones expressed greater levels of NTCP compared with the HepG2-NTCP-A3 parental clone (Figure 4A). To examine whether the relative expression of NTCP by HepG2-NTCP-A3 parental clone or its subclones correlated with HBV preS1 binding, we analyzed the binding of MyrB-Alexa-647 by fluorescence microscopy. The C2 and G7 subclones had the highest and the lowest MyrB binding compared to the HepG2-NTCP-A3 parental clone, respectively, and the differences were statistically significant (Figure 4B,C). In addition, no morphological or growth differences between the subclones and the parental clone were observed, indicating that the alteration in the NTCP expression levels were independent of morphology and growth differences. Further, the NTCP expression and permissiveness to HBV infection were only moderately correlated, implying that additional cellular factors, independent of NTCP expression, may influence HBV permissiveness (Figure 4D).

## 4. Discussion

The discovery of NTCP as a functional receptor for HBV has allowed the development of cell culture-based models suitable for the study of the HBV life cycle. Thus, the HepG2-NTCP cells provide a convenient infection model for the HBV infection that has been used extensively for drug development studies [4,5]. Given that NTCP is a bona fide receptor for HBV, any variation in its expression may affect the outcome and kinetics of HBV infection. Such an observation in our experimental setting prompted us to investigate the clonality of the HepG2-NTCP-A3 cell line. Using limiting dilution, we obtained multiple HepG2-NTCP-A3 subclones with varying degrees of permissiveness to HBV, suggesting that the parental HepG2-NTCP-A3 is indeed a mixed population of functionally distinct phenotypes (Figure 1A–C).

The low levels of NTCP expression are known to render hepatocytes poorly permissive to HBV infection [24]. It has also been shown that enhanced HBV infection in HepG2-NTCP cells is NTCP-dependent [19]. We observed that the NTCP expression differed between the subclones and the parental HepG2-NTCP-A3 (Figure 4A). We further showed that the NTCP expression levels reflected by MyrB binding-determined-immunofluorescence were significantly higher only in the C2 subclone compared to the HepG2-NTCp-A3 parental clone (Figure 4B,C). However, the relationship between the NTCP expression and permissiveness was not uniformly observed across the other subclones, suggesting other cellular factors may be operative in the regulation of HBV entry or replication [16,25,26,27]. This finding agrees with a previous report where the NTCP-expressing subclones also showed a similar binding affinity towards MyrB [16] suggesting that the intracellular NTCP might be playing a role in promoting HBV infection. Alternatively, a non-receptor role of NTCP in promoting HBV infection has also been suggested [28]. The original parental HepG2-NTCP-A3 clone was generated in 2014, through lentiviral-mediated transduction [12]; it is possible that successive passaging has altered its cellular characteristics and thus has affected its overall permissiveness to HBV. The understanding of the non-receptor function(s) of NTCP is limited and requires further investigation.

The addition of PEG is not physiologically relevant to human infection, as it creates a non-specific cell membrane fusion [20]. A total of 4% PEG is usually added to the incubation medium and is known to enhance the HBV entry process by facilitating the viral attachment to the cell-surface heparan sulfate proteoglycans [20,29,30]. The addition of PEG significantly enhanced the HBV infection for all of the clones, which is in line with previous reports [17,19,20]; however, the C2 subclone performed better than all of the other reported clones and the HepG2-NTCP-A3 parental clone in supporting PEG-free HBV infection [19]. In addition, in the presence of 4% PEG, the higher susceptibility of the C2 subclone to HBV correlated with the increased number of cccDNA copies (Figure 1A,D). However, attempts to quantify the cccDNA under PEG-free conditions were unsuccessful by qPCR, probably due to the lower infection percentage (data not shown). Indeed, the difference in permissiveness between the C2 and the other clones was greater in the PEG-free conditions than in the presence of PEG (Figure 2A–C; Table 1). Thus, the C2 subclone could be utilized for PEG-free HBV infection studies and it is likely that additional subcloning might further optimize the reduced dependency on PEG, which would be a significant advance.

In addition to enhancing the infection of the cell culture-derived HBV genotype D, the C2 subclone also showed enhanced infectivity with patient-derived HBV samples of genotype B and E, relative to the HepG2-NTCP-A3 parental clone. Interestingly, both the HepG2-NTCP-A3 parental clone as well as the C2 subclone showed reduced susceptibility to the genotype C patient-derived serum. The cause for the low infectivity with genotype C HBV is not clear, however similar observations of low infectivity of genotype C were attributed to the presence of a large proportion of non-infectious virus particles or the low infectivity of genotype C [16,31]. Since the primer pair 1040–1996 is specific for the formed cccDNA in genotype D, as described [22], we did not quantify the cccDNA in the patient-derived HBV samples (non-D genotype HBV), however based on the performance of the C2 subclone, it is tempting to speculate that the C2 subclone would show a higher number of cccDNA copies upon infection with other HBV genotypes.

Although the difference in cell growth could affect the outcome of HBV infection, we did not observe any difference in cell growth or in cellular morphology in any of the HepG2-NTCP-A3 subclones (Figure 4B). In a recent report, a slow-growing HepG2-NTCP-Sec^+^ subclone which requires high viral inoculum of up to 5000 GEq/mL, was shown to possess amplified HBV infectivity [16]. However, considering the practical disadvantages of slow growth and the requirement of high inoculum, the subclones described in the current study, especially C2, are advantageous and provide a more efficient HBV infection model. Moreover, previously reported growth differences in the HepG2-NTCP subclones, and observations of the varying permissiveness reported in the current study, suggest that functional heterogeneity exists within the HepG2-NTCP cells [16]. A similar heterogeneity has been reported for Huh7 cells [32], another hepatoma cell line that is susceptible to HBV infection upon exogenous NTCP expression [30].

In conclusion, we demonstrate that the enhancement of permissiveness through HepG2-NTCP subcloning provides an improved model for HBV infection studies. Since the HepG2-NTCP cells are also used for the assessment of novel HBV antivirals, our findings suggest that the hepatoma cell lines expressing NTCP should be routinely monitored for any variation in NTCP expression, or permissiveness to HBV, when studying the HBV life cycle.

## Figures and Tables

**Table 1:** Comparison of HBV infection in the presence or absence of PEG in HepG2-NTCP parental clone and its subclones.

	4% PEG				No Peg			
	Extracellular HBV DNA		Intracellular HBV DNA		Extracellular HBV DNA		Intracellular HBV DNA	
	HBV DNA log_10_Copies/mL (SD)	Fold Change vs. Parental	HBcAg MFI	Fold Change vs. Parental	HBV DNA log_10_Copies/mL (SD)	Fold Change vs. Parental	HBV DNA log_10_Copies/mL (SD)	Fold Change vs. Parental
HepG2-NTCP-A3	6.20 ± 5.52	1.0	69.1 ± 1.3	1.0	4.21 ± 2.15	1.0	16.1 ± 0.4	1.0
B7	6.66 ± 4.94 ^a^	2.98	84.7 ± 2.6	1.22	4.43 ± 3.45 ^b^	1.67	16.7 ± 0.4	1.03
C2	6.84 ± 5.93 ^a^	4.45	93.0 ± 2.3 ^a^	1.35	4.77 ± 2.51 ^b^	3.63	24.7 ±1.5 ^b^	1.48
D10	6.65 ± 4.06 ^a^	2.80	79.6 ± 1.3	1.15	4.42 ± 2.43 ^b^	1.63	20.4 ± 0.6	1.22
G4	6.59 ± 4.07 ^a^	2.57	77.3 ± 1.5	1.12	4.08 ± 2.17	0.74	16.6 ± 0.9	0.99
G7	6.54 ± 5.37 ^a^	2.25	74.6 ± 1.5	1.08	4.03 ± 2.79	0.67	10.1 ± 4.1	0.60

^a^ Significantly different from 4% PEG control (HepG2-NTCP-A3) (*p* < 0.05); ^b^ Significantly different from No PEG control (HepG2-NTCP-A3) (*p* < 0.05).

## Data Availability

Not applicable.
